# Comparative Perspectives on Obesity From Medical Students, Practitioners, and Non-medical Students

**DOI:** 10.7759/cureus.114546

**Published:** 2026-08-14

**Authors:** Sophia Rafiq, Zainab Pervaiz, Taleah Khan, Abdullah Ahmad, Amna Nadeem

**Affiliations:** 1 Internal Medicine, Institute of Dentistry, Combined Military Hospital (CMH) Lahore Medical College, Lahore, PAK; 2 Pediatrics, Institute of Dentistry, Combined Military Hospital (CMH) Lahore Medical College, Lahore, PAK; 3 Physiology, Institute of Dentistry, Combined Military Hospital (CMH) Lahore Medical College, Lahore, PAK

**Keywords:** obesity, obesity and nutrition, obesity and overweight, obesity attitude, obesity knowledge, obesity medication, perception, weight perception, weight stigma

## Abstract

Objective: Obesity is a multifactorial chronic disease with a rising prevalence globally and in Pakistan. Despite this, public understanding and acceptance of obesity as a disease remain inconsistent, often contributing to stigma and delayed care. Examining how different groups perceive obesity is essential for guiding public health efforts and reducing weight-related stigma.

Methods: This comparative analytical cross-sectional study included 264 participants recruited through convenience sampling between November and December 2025 from Combined Military Hospital (CMH) Lahore Medical College and Lahore University of Management Sciences (LUMS). Data were collected using a questionnaire adapted from the 33-item survey by Puhl and Liu assessing perceptions of obesity as a disease and attitudes toward its classification and treatment. Following assessment of normality, non-normally distributed variables were analyzed using the Kruskal-Wallis test, while normally distributed variables were compared using ANOVA with post hoc analysis, with statistical significance set at p ≤ 0.05.

Results: There were 264 participants in total, including medical professionals, medical students, and non-medical students. The majority were female (72.0%), aged between 18 and 24 (56.1%), and aware of body mass index (96.6%). Regarding the factors supporting the classification of obesity as a disease, there was no discernible difference between the three groups (Kruskal-Wallis, p = 0.436). On the other hand, the opposing elements showed a significant difference (ANOVA, p = 0.036). While no significant differences were found between the other group comparisons, post hoc analysis showed that medical practitioners had significantly lower opposing scores than non-medical students (p = 0.041).

Conclusion: Medical training and clinical exposure were associated with greater recognition of obesity as a disease and lower levels of stigmatizing attitudes. These findings highlight the need for educational interventions and culturally relevant awareness programs to improve understanding of obesity and address stigma in broader populations, especially in South Asia.

## Introduction

Obesity is a complex, multifactorial condition characterized by excessive adiposity that impairs health. Body mass index (BMI), calculated as weight in kilograms divided by height in meters squared, remains the most widely used measure for classifying weight status, with obesity defined as BMI ≥30 kg/m² and overweight as BMI 25-29.9 kg/m² [1]. According to the World Health Organization (WHO), as of 2022, around 2.5 billion adults aged 18 years and older were overweight, with 890 million living with obesity. This accounts for 43% of adults being overweight and 16% were living with obesity worldwide. The global prevalence of obesity has more than doubled since 1990, reflecting a significant and growing public health concern [2]. This trend extends to children as well. In 2022, 390 million children and adolescents aged 5 to 16 were overweight, and by 2024, 35 million children under the age of five were overweight [2]. Alarmingly, projections suggest that by 2030, over five million school-aged children in Pakistan will be affected by obesity [3].

The implications are substantial, as obesity contributes to the rising burden of non-communicable diseases such as type 2 diabetes, cardiovascular disease, and hypertension, as well as over 13 types of cancer, including colorectal, breast, and pancreatic cancers [4,5]. Lifestyle changes, poor dietary habits, and increasing urbanization have further fueled this trend [6]. Even though the Pakistani population generally views obesity negatively, in some regions, it is undeservedly glorified and perceived as a sign of affluence [7].

Despite global recognition of obesity as a chronic disease, public understanding and perceptions remain inconsistent across different population groups. Studies have shown that medical students may continue to exhibit implicit and explicit weight bias despite medical training, while public views on obesity classification are associated with beliefs about personal responsibility, stigma, and treatment approaches [8,9]. Furthermore, disease-framing messages regarding obesity may be associated with differences in attitudes toward its management and self-regulation [10]. Although obesity is increasingly recognized as a chronic disease in the Asia-Pacific region, existing evidence has primarily examined perceptions among individuals with obesity and healthcare professionals. A multi-country survey across the region found that, despite high recognition of obesity as a disease among both groups, misconceptions regarding responsibility for weight management and treatment preferences remained common [11].

Existing studies in Pakistan and South Asia have primarily focused on obesity prevalence, associated risk factors, and clinical management, with limited investigation of differences in obesity-related perceptions between medical and non-medical groups [7]. Therefore, this study aims to compare perceptions of obesity as a disease among medical practitioners, medical students, and non-medical students. By identifying variations in understanding and attitudes across these groups, this study may help guide targeted educational initiatives, support efforts to address obesity-related stigma, and inform culturally relevant public health interventions.

## Materials and methods

This cross-sectional study was conducted at Combined Military Hospital (CMH) Lahore Medical College, Lahore University of Management Sciences, and the Combined Military Hospital in Lahore, Pakistan, from November to December 2025, with approval from the Institutional Ethics Review Committee (ERC) (Case no. 160/ERC/CMH/LMC). 

The required sample size was calculated using Cochran’s formula for cross-sectional studies to estimate a population proportion (n = Z^2^(1−α/2) × P(1−P)​/e^2^), where Z = 1.96 represents the standard normal deviate for a 95% confidence level. The expected proportion was set at 60% (p = 0.60) based on the findings of the parent study [9], which assessed perceptions regarding obesity as a disease. With a margin of error (precision) of 6% (e = 0.06), the minimum required sample size was calculated to be 256 participants, who were recruited through convenience sampling.

Eligible participants were adults aged ≥18 years and were categorized into three groups: (1) medical practitioners with a minimum of five years of clinical experience, (2) medical students enrolled from first to final year, and (3) non-medical students currently enrolled in a bachelor’s program in a non-medical field. Individuals were excluded if they were taking medications known to influence weight, including antidepressants, antiepileptics, antipsychotics, antihypertensive agents, antidiabetic drugs, or hormonal contraceptives [12]. Participants with polycystic ovary syndrome or hypothyroidism were also excluded [13,14].

The prespecified primary outcome was the difference in obesity-related perceptions among medical practitioners, medical students, and non-medical students. The study assessed whether the level of medical training and clinical exposure were associated with variations in perceptions of obesity as a chronic disease.

Data were collected using a validated questionnaire developed by Puhl and Liu, originally used in a national survey examining public views on classifying obesity as a disease [9]. The questionnaire consisted of 33 statements, including 17 statements supporting the classification of obesity as a disease and 16 statements opposing this classification. All statements were presented in a random order. Each statement was scored from 1 to 5 using a Likert scale, with responses ranging from Strongly Disagree (score 1), Disagree (score 2), Neutral (score 3), Agree (score 4), to Strongly Agree (score 5) (Appendix).

Consistent with the approach used by Puhl and Liu [9], the statements were interpreted as two domains: statements supporting the classification of obesity as a disease (17 statements) and statements opposing this classification (16 statements). Separate scores were calculated for each domain. Part 1 consisted of 17 statements supporting obesity as a disease, with a maximum score of 85 and a minimum score of 17, while Part 2 consisted of 16 statements opposing this classification, with a maximum score of 80 and a minimum score of 16. The questionnaire was administered after obtaining informed and voluntary consent from each participant. All data were handled confidentially and in accordance with the principles outlined in the Declaration of Helsinki.

Data entry and statistical analysis were performed using IBM SPSS Statistics for Windows, Version 21.0 (Released 2012; IBM Corp., Armonk, NY, USA) [15]. The normality of the data was assessed using the Kolmogorov-Smirnov test. Data of Part 1 were non-normal; therefore, the Kruskal-Wallis test was applied. Data of Part 2 were normally distributed; therefore, one-way ANOVA was applied for intergroup comparisons, followed by Bonferroni-adjusted post hoc pairwise comparisons. Quantitative variables were summarized as mean ± standard deviation and percentages. A p-value of ≤0.05 was considered statistically significant.

## Results

A total of 264 participants were included in the study. The mean age of the participants was 27.55 years, with more than half of the sample aged 18-24 years (148 [56.1%]). The majority were female (190 [72.0%]), while 74 (28.0%) were male. Female participants were predominant among medical students (80 [94.1%]) and medical practitioners (67 [75.3%]), whereas the non-medical student group had a more balanced gender distribution (43 females and 47 males). A total of 165 (62.5%) participants reported having a doctor in their immediate family, while 99 (37.5%) did not. Awareness of body mass index (BMI) was high, with 255 (96.6%) participants being aware and 9 (3.4%) being unaware. Additionally, 218 (82.6%) participants reported having an overweight or obese individual in their close circle, compared to 46 (17.4%) who did not. The baseline characteristics of the participants across the three groups are summarized in Table 1.

**Table 1:** Group-wise baseline demographic characteristics of study participants. Data are presented as n (%). Percentages within the medical practitioner, medical student, and non-medical student groups represent the proportion of participants within each respective group. Percentages in the total column represent the proportion of the overall study population (n = 264). BMI: body mass index.

Characteristics	Medical practitioners, n = 89	Medical students, n = 85	Non-medical students, n = 90	Total, n = 264
Gender				
Female	67 (75.3%)	80 (94.1%)	43 (47.8%)	190 (72.0%)
Male	22 (24.7%)	5 (5.9%)	47 (52.2%)	74 (28.0%)
Age category				
18-24	0	73 (85.6%)	75 (83.3%)	148 (56.1%)
25-29	32 (36.0%)	12 (14.1%)	15 (16.7%)	59 (22.3%)
30-34	13 (14.6%)	0	0	13 (4.9%)
35-49	21 (23.6%)	0	0	21 (8.0%)
50+	23 (25.8%)	0	0	23 (8.7%)
Doctors in immediate family				
Yes	65 (73.0%)	42 (49.4%)	58 (64.4%)	165 (62.5%)
No	24 (27.0%)	43 (50.6%)	32 (35.6%)	99 (37.5%)
Awareness of BMI				
Yes	88 (98.9%)	83 (97.6%)	84 (93.3%)	255 (96.6%)
No	1 (1.1%)	2 (2.4%)	6 (6.7%)	9 (3.4%)
Family or close circle member overweight/obese				
Yes	76 (85.4%)	73 (85.9%)	69 (76.7%)	218 (82.6%)
No	13 (14.6%)	12 (14.1%)	21 (23.3%)	46 (17.4%)

A total of 264 participants were included in the study, comprising 89 medical practitioners, 85 medical students, and 90 non-medical students. The questionnaire comprised 33 items, with 17 supporting the classification of obesity as a disease (Part 1) and 16 opposing the classification (Part 2). The frequencies and proportions of participants who agreed or strongly agreed with statements supporting the classification of obesity as a disease (Part 1) are presented in Table 2.

**Table 2:** Agreement (agree + strongly agree) with statements supporting the classification of obesity as a disease across the three groups. ^a^Agreement was defined as endorsement of "4" (agree) or "5" (strongly agree) on the 5-point Likert rating scale.

Statement (Agreement^a^)	Medical practitioners, n = 89	Medical students, n = 85	Non-medical students, n = 90
Classifying obesity as a disease will lead more doctors to spend time talking to patients about their weight	44 (49.4%)	40 (47.1%)	46 (51.1%)
Labeling obesity as a disease is an important step in helping people gain access to obesity treatment	72 (80.9%)	61 (71.8%)	69 (76.7%)
Classifying obesity as a disease will help ensure that more resources are dedicated to research for obesity prevention and treatment	60 (67.4%)	51 (60.0%)	63 (70.0%)
There is a medical basis for obesity to be classified as a disease, because like other diseases obesity may impair the normal functioning of a body, decrease life expectancy, or cause death	53 (59.6%)	55 (64.7%)	60 (66.7%)
Recognizing obesity as a disease will lead medical professionals to treat obesity more seriously	42 (47.2%)	40 (47.1%)	44 (48.9%)
Classifying obesity as a disease will help improve reimbursement for weight loss counseling and education	60 (67.4%)	51 (60.0%)	65 (72.2%)
Classifying obesity as a disease will lead to better medical care for people with obesity	67 (75.3%)	58 (68.2%)	61 (67.8%)
Labeling obesity as a disease is an important step in acknowledging the complexity of this particular condition	70 (78.7%)	61 (71.8%)	63 (67.8%)
Recognizing obesity as a disease will improve the way that the medical community addresses this health issue	53 (59.6%)	54 (63.5%)	61 (70.0%)
Classifying obesity as a disease will improve insurance reimbursements for obesity drugs, weight loss surgery, and counseling	60 (67.4%)	50 (58.8%)	66 (73.3%)
The classification of obesity as a disease will encourage insurance companies to provide better coverage of obesity treatment	60 (67.4%)	50 (58.8%)	64 (71.1%)
By labeling obesity as a disease, doctors will address obesity with more compassion and respect	53 (59.6%)	55 (64.7%)	61 (67.8%)
Obesity meets the medical definition of ‘disease’ in that it is a physiological dysfunction of the human organism with environmental, genetic, and endocrinological causes	67 (75.3%)	53 (62.4%)	53 (58.9%)
Classifying obesity as a disease will reduce the stigma experienced by obese individuals, who are often blamed for their condition	36 (40.4%)	24 (28.2%)	23 (25.6%)
Obesity is not a matter of poor willpower or laziness and should be treated like a disease	67 (75.3%)	56 (65.9%)	60 (66.7%)
Obesity should be classified as a disease because the social benefits of doing so will outweigh the costs	70 (78.7%)	59 (69.4%)	64 (71.1%)
Obesity should be classified as a disease	67 (75.3%)	49 (57.6%)	45 (50.0%)

Overall, respondents from all three groups expressed high levels of agreement with many of the supporting statements. Medical practitioners consistently demonstrated greater agreement with the fundamental concept of obesity as a disease than the other two groups. Specifically, 75.3% (67/89) of medical practitioners agreed that obesity should be classified as a disease, compared with 57.6% (49/85) of medical students and 50.0% (45/90) of non-medical students. Likewise, agreement that obesity meets the definition of a physiological dysfunction was highest among medical practitioners (67/89, 75.3%), followed by medical students (53/85, 62.4%) and non-medical students (53/90, 58.9%). These findings suggest that medical practitioners were more likely than both student groups to endorse obesity as a disease based on its underlying pathophysiology.

Among medical practitioners, the highest levels of agreement were for the statements that labeling obesity as a disease is an important step in helping people gain access to obesity treatment (72/89, 80.9%), labeling obesity as a disease is an important step in acknowledging the complexity of obesity (70/89, 78.7%), and obesity should be classified as a disease because the health benefits outweigh the costs (70/89, 78.7%) (Table 2).

Similarly, among medical students, the greatest agreement was observed for improving access to obesity treatment (61/85, 71.8%), acknowledging the complexity of obesity (61/85, 71.8%), and obesity should be classified because the health benefits outweigh the costs (59/85, 69.4%) (Table 2).

Among non-medical students, the highest agreement was for the statements that classifying obesity as a disease would improve reimbursement for weight loss counselling and education (65/90, 72.2%), obesity should be classified because the health benefits outweigh the costs (64/90, 71.1%), and classification would increase resources dedicated to obesity research (63/90, 70.0%) (Table 2).

Across all three groups, the lowest agreement was for the statement that classifying obesity as a disease would reduce the stigma experienced by individuals living with obesity, with agreement from 36 (40.4%) medical practitioners, 24 (28.2%) medical students, and 23 (25.6%) non-medical students. Agreement was also relatively low for the statement that recognizing obesity as a disease would lead medical professionals to treat obesity more seriously, with 42/89 (47.2%), 40/85 (47.1%), and 44/90 (48.9%) participants agreeing, respectively (Table 2). Overall, supporting element scores are summarized in Table 3.

**Table 3:** Mean, median, and range scores of statements supporting classification of obesity as a disease across all three groups.

Group	Mean ± SD	Median (IQR)	Range
Medical practitioners	58.69 ± 6.00	59 (55–63)	43-71
Medical students	59.10 ± 6.99	60 (56- 63)	29-73
Non-medical students	59.39 ± 6.63	60 (56.75–64)	36-71

Medical practitioners had a mean score of 58.69 ± 6.00 (median = 59, IQR = 55-63), medical students had a mean score of 59.10 ± 6.99 (median = 60, IQR = 56-63), and non-medical students had a mean score of 59.39 ± 6.63 (median = 60, IQR = 56.75-64). A comparison of supporting element scores using the Kruskal-Wallis test demonstrated no statistically significant difference among the three groups (H = 1.66, p = 0.436), indicating similar overall agreement with statements supporting obesity as a disease (Table 4).

**Table 4:** Kruskal-Wallis test for supporting elements (Part 1). ^a^Significance was set at p-value <0.05.

Supporting elements	Group categories	Median (IQR = Q3-Q1)	Mean rank	Kruskal-Wallis	p-value^a^
	Medical practitioner (n = 89)	60 (63-56)	124.23	1.66	0.436
	Medical student (n = 85)	63 (63-55)	133.76		
	Non-medical students (n = 90)	60 (64-56.75)	138.97		
	Total N = 264				

The frequencies and proportion of participants who agreed or strongly agreed with statements opposing the classification of obesity as a disease (Part 2) are presented in Table 5.

**Table 5:** Agreement (agree + strongly agree) with statements opposing the classification of obesity as a disease across the three groups. ^a^Agreement was defined as endorsement of "4" (agree) or "5" (strongly agree) on the 5-point Likert rating scale.

Statement (Agreement^a^)	Medical practitioners, n = 89	Medical students, n = 85	Non-medical students, n = 90
Recognizing obesity as a disease, as opposed to a "condition" or "disorder" will not necessarily result in improved health outcomes for patients	17 (19.1%)	15 (17.6%)	9 (10.0%)
Classifying obesity as a disease will lead medical professionals to rely too much on medications and surgery to treat obesity	42 (47.2%)	40 (47.1%)	44 (48.9%)
Labeling obesity as a disease will shift the focus away from measures to encourage healthy diets and regular exercise	33 (37.1%)	48 (56.5%)	59 (65.6%)
Classifying obesity as a disease will make more employers reluctant to hire obese employees	11 (12.4%)	13 (15.3%)	13 (14.4%)
Classifying obesity as a disease will shift focus away from important environmental factors that contribute to obesity (such as fast food restaurants, marketing of unhealthy foods, the cost of unhealthy versus healthy foods)	72 (80.9%)	61 (71.8%)	74 (82.2%)
Obesity should not be considered a disease because obesity is more a risk factor for other conditions (e.g., like diabetes and heart disease) rather than a disease in its own right	26 (29.2%)	35 (41.2%)	35 (38.9%)
Labeling obesity as a disease will lead doctors to rely too much on a patient’s body weight to determine his/her health status	45 (50.6%)	50 (58.8%)	51 (56.7%)
Obesity should not be classified as a disease, because not all obese persons develop diabetes or heart disease, suggesting that obesity alone is a poor predictor of health	17 (19.1%)	14 (16.5%)	10 (11.1%)
Obesity should not be considered a disease because the main measure to determine obesity is body mass index (BMI), which is simplistic and flawed	40 (44.9%)	31 (36.5%)	34 (37.8%)
Labeling obesity as a disease will not change the way that doctors see their patients	45 (50.6%)	50 (58.8%)	52 (57.8%)
Obesity has been classified as a disease mostly in response to new FDA-approved drugs for weight loss	36 (40.4%)	39 (45.9%)	17 (18.9%)
Since obesity does not always lead to poor health (e.g., some obese persons are fit and eat healthy), obesity should not be considered a disease	17 (19.1%)	15 (17.6%)	9 (10.0%)
Since so many diseases are difficult to cure, labeling obesity as a disease will lead obese individuals to feel pessimistic about improving their health	32 (36.0%)	46 (54.1%)	37 (41.1%)
Obesity should not be considered a disease because there are no specific symptoms associated with obesity	44 (49.4%)	48 (56.5%)	50 (55.6%)
Classifying obesity as a disease will increase the stigma and discrimination experienced by obese individuals	35 (39.3%)	44 (51.8%)	44 (48.9%)
There is no agreed-upon scientific definition of ‘disease,’ so obesity should not be classified as a disease	17 (19.1%)	14 (16.5%)	11 (12.2%)

Comparisons between the three groups showed notable differences in perceptions opposing obesity as a disease. Medical students (41.2%) and non-medical students (38.9%) were more likely than medical practitioners (29.2%) to agree that obesity is mainly a risk factor rather than a disease. Medical students also showed the greatest agreement that disease labeling leads to patient pessimism (46/85, 54.1%), compared with non-medical students (37/90, 41.1%) and medical practitioners (32/89, 36.0%). Similarly, agreement that disease labeling promotes medication over lifestyle changes increased progressively across the groups, from medical practitioners (33/89, 37.1%) to medical students (48/85, 56.5%) and was highest among non-medical students (59/90, 65.6%). In contrast, agreement that obesity has been classified as a disease in response to the availability of weight loss drugs such as semaglutide was similar among medical practitioners (36/89, 40.4%) and medical students (39/85, 45.9%) but substantially lower among non-medical students (17/90, 18.9%).

The statement with the highest agreement across all three groups was that classifying obesity as a disease would shift attention away from important environmental contributors to obesity, with agreement from 72/89 (80.9%) medical practitioners, 61/85 (71.8%) medical students, and 74/90 (82.2%) non-medical students (Table 5).

Moderately high agreement was also observed for the statements that labeling obesity as a disease would lead doctors to rely excessively on patients' body weight when assessing health, with agreement from 45/89 (50.6%) medical practitioners, 50/85 (58.8%) medical students, and 51/90 (56.7%) non-medical students, and that labeling obesity as a disease would not change the way doctors view their patients, with agreement from 45/89 (50.6%), 50/85 (58.8%), and 52/90 (57.8%), respectively (Table 5).

Conversely, the lowest agreement was observed for the statement that classifying obesity as a disease would make employers more reluctant to hire individuals living with obesity, with agreement from 11/89 (12.4%) medical practitioners, 13/85 (15.3%) medical students, and 13/90 (14.4%) non-medical students. Similarly, few participants agreed that obesity should not be classified as a disease because not all individuals with obesity develop diabetes or cardiovascular disease (17/89 [19.1%], 14/85 [16.5%], and 10/90 [11.1%], respectively) or that there is no agreed-upon scientific definition of disease (17/89 [19.1%], 14/85 [16.5%], and 11/90 [12.2%], respectively) (Table 5). Overall, opposing element scores are summarized in Table 6.

**Table 6:** Mean, median, and range scores of statements opposing the classification of obesity as a disease across all three groups.

Group	Mean ± SD	Median (IQR)	Range
Medical practitioners	50.55 ± 6.66	51 (46-55)	34-70
Medical students	52.34 ± 6.18	52 (49-56.25)	34-67
Non-medical students	52.84 ± 5.45	53 (50-57)	34-65

Medical practitioners had the lowest mean opposing score (50.55 ± 6.66; median = 51, IQR = 46-55), followed by medical students (52.34 ± 6.18; median = 52, IQR = 49-56.25), while non-medical students had the highest mean score (52.84 ± 5.45; median = 53, IQR = 50-57). A one-way analysis of variance demonstrated a statistically significant difference in opposing element scores among the three groups (F(2,261) = 3.37, p = 0.036) (Table 7).

**Table 7:** Comparison between the three groups for opposing elements (Part 2). ^*^Statistically significant at p < 0.05.

Opposing part	Sum of squares	df	Mean square	F	p-value
Between groups	251.19	2	125.60	3.37	0.036*
Within groups	9722.95	261	37.25		
Total	9974.15	263			

Bonferroni-adjusted post hoc analysis demonstrated a statistically significant difference only between medical practitioners and non-medical students (mean difference = −2.30, 95% CI: −4.53 to −0.07, adjusted p = 0.041). No significant differences were observed between medical practitioners and medical students (adjusted p = 0.160) or between medical students and non-medical students (adjusted p = 1.000) (Table 8).

**Table 8:** Bonferroni-adjusted post hoc test for multiple factors of opposing elements (Part 2). ^a^Statistical significance set at p < 0.05. ^b^Extracted p-values are Bonferroni-adjusted. ^*^Statistically significant.

Group categories		Difference ± SD error	p-value^a,b^	95% Confidence interval	
				Lower bound	Upper bound
Medical practitioners	Medical students	-1.80 ± 0.926	0.160	-4.02	0.4343
	Non-medical students	-2.30 ± 0.926	0.041*	-4.528	-0.0657
Medical students	Non-medical students	-0.50 ± 0.910	1.00	-2.692	1.6923

Overall, while the three groups demonstrated comparable levels of agreement with statements supporting the classification of obesity as a disease, significant differences were observed for opposing element scores. Non-medical students expressed greater agreement with statements opposing obesity as a disease than medical practitioners, whereas medical students did not differ significantly from either group.

## Discussion

Obesity is increasingly recognized as a chronic, multifactorial disease shaped by complex biological, environmental, and psychosocial determinants [6]. Despite this growing consensus, perceptions of obesity remain heterogeneous and are strongly influenced by educational background and clinical exposure. These perceptions may be associated with differences in stigma, health-seeking behaviors, and treatment approaches. This study demonstrates differences in obesity conceptualization among medical practitioners, medical students, and non-medical students, highlighting the association between educational background, professional experience, and beliefs about obesity.

This study evaluated attitudes toward the classification of obesity as a disease among medical practitioners, medical students, and non-medical students. The results showed that although participants in all three groups agreed with comments endorsing obesity as an illness at similar levels, there were disparities when it came to opposing views.

The clinical and public health relevance of these findings is substantial, given the well-established association between obesity and a wide range of adverse health outcomes, including obstructive sleep apnea, chronic kidney disease, polycystic ovary syndrome, stroke, coronary artery disease, and an increased risk of certain malignancies such as breast and colorectal cancer [6,16]. Beyond its physical consequences, obesity is associated with psychosocial burdens, including weight bias and stigma, which may contribute to low self-esteem, depression, social withdrawal, and internalized weight stigma [8,17,18]. Prior research has shown that self-stigma is associated with poorer dietary adherence, reduced motivation, and lower self-efficacy for engaging in health-promoting behaviors, thereby perpetuating the cycle of obesity [18,19]. In this context, understanding how obesity is perceived, particularly by healthcare professionals, is essential for improving patient care and reducing stigma.

In this study, these results imply that regardless of professional or educational background, support for the diagnosis of obesity as an illness was largely constant. Similar views among healthcare professionals and the general public may reflect the growing visibility of obesity through public health campaigns, increased awareness, and extensive media coverage. This gradient mirrors findings from previous literature suggesting that clinicians are more likely to adopt a disease-based framework for obesity due to greater exposure to evidence linking obesity with neurohormonal dysregulation, genetic susceptibility, and metabolic dysfunction [20]. But in another past study, medical students showed an intermediate position, indicating that while medical education introduces the biological basis of obesity, it may not fully translate into firm acceptance of obesity as a chronic disease [8].

In a similar way, polls conducted after professional associations recognized obesity as an illness showed growing acceptance among students and healthcare professionals. Medical professionals and students demonstrated levels of agreement similar to those of non-medical students, which may be explained by improved medical knowledge of obesity pathogenesis. Similar perceptions have been reported in earlier studies, in which obesity is often framed as a consequence of individual lifestyle choices rather than as a chronic condition requiring long-term management [21,22]. Such views may be associated with greater attribution of blame and lower acceptance of medical interventions. By contrast, recognizing obesity as an independent disease aligns with contemporary clinical guidelines that emphasize early intervention irrespective of comorbidities [23,24].

Comprehensive lifestyle programs incorporating diet, physical activity, and behavioral therapy remain central to obesity management and have demonstrated effectiveness in improving weight and cardiometabolic outcomes [25]. These findings highlight the need for public messaging that clarifies that medical treatment for obesity complements, rather than replaces, lifestyle changes and personal effort [26].

Opposing attitude scores varied significantly among the three groups, whereas supportive aspects did not (ANOVA, p = 0.036). According to the post hoc analysis, there was only a significant difference (p = 0.041) between medical practitioners and non-medical students; there were no significant differences between medical students and non-medical students or between medical practitioners and medical students. According to these results, medical professionals were less likely than non-medical students to support claims that obesity is a disease. Their increased clinical exposure and comprehension of obesity as a chronic illness may lessen the perception that personal lifestyle decisions are the exclusive cause of obesity. Recognizing obesity as a complicated medical problem requiring all-encompassing care may also be reinforced by regular interactions with patients who are dealing with obesity-related comorbidities.

Persky and Eccleston [27], who found that more medical education was linked to more evidence-based conceptions of obesity and less reliance on oversimplified explanations emphasizing personal responsibility alone, corroborate this observation. Comparably, research by Puhl and Liu revealed that, in comparison with people without healthcare training, healthcare professionals who had received education about obesity showed less agreement with stigmatizing ideas and more acceptance of disease classification [28].

From a public health perspective, the findings are particularly relevant in South Asian contexts, where obesity is often simultaneously stigmatized and, in some settings, socially normalized or even perceived positively as an indicator of wealth and well-being [29]. Misconceptions surrounding obesity may delay care-seeking and exacerbate health inequities. By demonstrating associations between educational background, clinical exposure, and obesity perceptions, this study supports the need for targeted awareness initiatives, not only within medical institutions but also in schools and community settings. Such efforts should emphasize the multifactorial nature of obesity while promoting supportive, stigma-free environments.

This study has several strengths, including the inclusion of three distinct participant groups and the use of a previously published questionnaire. However, some limitations should be considered. First, convenience sampling from a limited number of institutions may restrict the generalizability of the findings and may have introduced institutional confounding, as differences between participating institutions could have influenced participants' perspectives. Second, because the study was cross-sectional, it captures perceptions at a single point in time and cannot establish causal relationships. In addition, responses were self-reported and may have been influenced by social desirability bias, with participants providing answers they perceived as more acceptable. The predominance of female participants (72%) represents a demographic imbalance that may have influenced the findings, as gender-related differences in health perceptions and attitudes cannot be excluded. Furthermore, the analyses were not adjusted for participants' personal BMI or obesity status, which may have affected their perceptions of obesity.

Despite these limitations, this study provides valuable insights into the perceptions of obesity among medical practitioners, medical students, and non-medical students. Future studies employing probability sampling, recruiting participants from a wider range of institutions, adjusting for potential confounders such as BMI and obesity status, and using longitudinal designs are needed to better understand how perceptions of obesity evolve over time and influence clinical practice and health-related behaviors.

## Conclusions

The study found that medical professionals, medical students, and non-medical students all had generally favorable opinions of acknowledging obesity as a disease. Regarding the factors that support the obesity as a disease, all groups showed comparable degrees of agreement. The opposing elements did, however, differ, with medical professionals exhibiting noticeably less antagonism than non-medical students. These results suggest that views of obesity as a chronic illness may be associated with clinical experience and medical education. Even while disease classification is generally accepted, there are still some misunderstandings and misgivings, especially among people who are not medical professionals. Promoting accurate knowledge about obesity across all sectors of society, especially in culturally diverse contexts like South Asia, is essential to support effective public health strategies and foster empathetic attitudes toward individuals living with obesity.

## Appendices

Questionnaire: perceptions of obesity as a disease

The American Medical Association (AMA) recently announced that obesity is now officially classified as a "disease." Please indicate to what extent you agree or disagree with the following statements.

Strongly disagree

1

Disagree

2

Neither agree nor disagree

3

Agree

4

Strongly agree

5

1. Obesity meets the medical definition of "disease" in that it is a physiological dysfunction of the human organism with environmental, genetic and endocrinological causes.

              1                              2                              3                              4                              5

2. Classifying obesity as a disease will make more employers reluctant to hire obese employees.

             1                              2                              3                              4                              5

3. Obesity should not be considered a disease because the main measure to determine obesity is body mass index (BMI), which is too simplistic and flawed.

             1                              2                              3                              4                              5

4. Recognizing obesity as a disease, as opposed to a "condition" or "disorder," will not necessarily result in improved health outcomes for patients.

             1                              2                              3                              4                              5

5. There is no agreed-upon scientific definition of "disease," so obesity should not be classified as a disease.

             1                              2                              3                              4                              5

6. Classifying obesity as a disease will lead to better medical care for people with obesity.

             1                              2                              3                              4                              5

7. Obesity should not be considered a disease because obesity is more a risk factor for other conditions (e.g., like diabetes and heart disease) rather than a disease in its own right.

             1                              2                              3                              4                              5

8. Classifying obesity as a disease will improve insurance reimbursements for obesity drugs, weight loss surgery, and counseling.

             1                              2                              3                              4                              5

9. Classifying obesity as a disease will lead more doctors to spend time talking to patients about their weight.

             1                              2                              3                              4                              5

10. Labeling obesity as a disease is an important step in helping people gain access to obesity treatment.

             1                              2                              3                              4                              5

11. Labeling obesity as a disease will not change the way that doctors see their patients.

             1                              2                              3                              4                              5

12. Obesity is not a matter of poor willpower or laziness, and should be treated like a disease.

             1                              2                              3                              4                              5

13. Labeling obesity as a disease will shift the focus away from measures to encourage healthy diets and regular exercise.

            1                              2                              3                              4                              5

14. Obesity should not be considered a disease because there are no specific symptoms associated with obesity.

             1                              2                              3                              4                              5

15. Obesity should be classified as a disease because the social benefits of doing so will outweigh the costs.

             1                              2                              3                              4                              5

16. Recognizing obesity as a disease will improve the way that the medical community addresses this health issue.

             1                              2                              3                              4                              5

17. Labeling obesity as a disease is an important step in acknowledging the complexity of this particular condition.

             1                              2                              3                              4                              5

18. Classifying obesity as a disease will lead medical professionals to rely too much on medications and surgery to treat obesity.

             1                              2                              3                              4                              5

19. Classifying obesity as a disease will increase the stigma and discrimination experienced by obese individuals.

             1                              2                              3                              4                              5

20. Since so many diseases are difficult to cure, labeling obesity as a disease will lead obese individuals to feel pessimistic about improving their health.

             1                              2                              3                              4                              5

21. There is a medical basis for obesity to be classified as a disease, because like other diseases obesity may impair the normal functioning of a body, decrease life expectancy, or cause death.

             1                              2                              3                              4                              5

22. Classifying obesity as a disease will shift focus away from important factors in our society that contribute to obesity (such as fast food restaurants, marketing of unhealthy foods, the cost of unhealthy versus healthy foods).

             1                              2                              3                              4                              5

23. The classification of obesity as a disease will encourage insurance companies to provide better coverage of obesity treatment.

             1                              2                              3                              4                              5

24. Recognizing obesity as a disease will lead medical professionals to treat obesity more seriously.

             1                              2                              3                              4                              5

25. Classifying obesity as a disease will help improve reimbursement for weight loss counseling and education.

             1                              2                              3                              4                              5

26. By labeling obesity as a disease, doctors will address obesity with more compassion and respect.

             1                              2                              3                              4                              5

27. Classifying obesity as a disease will reduce the stigma experienced by obese individuals, who are often blamed for their condition.

             1                              2                              3                              4                              5

28. Since obesity does not always lead to poor health (e.g., some obese persons are fit and eat healthy), obesity should not be considered a disease.

             1                              2                              3                              4                              5

29. Obesity should not be classified as a disease, because not all obese persons develop diabetes or heart disease, suggesting that obesity alone is a poor predictor of health.

             1                              2                              3                              4                              5

30. Classifying obesity as a disease will help ensure that more resources are dedicated to research for obesity prevention and treatment.

             1                              2                              3                              4                              5

31. Obesity has been classified as a disease mostly in response to new FDA-approved drugs for weight loss.

             1                              2                              3                              4                              5

32. Labeling obesity as a disease will lead doctors to rely too much on a patient’s body weight to determine his/her health status.

             1                              2                              3                              4                              5

33. Obesity should be classified as a disease.

             1                              2                              3                              4                              5
